# Analyzing *Arabidopsis thaliana* root proteome provides insights into the molecular bases of enantioselective imazethapyr toxicity

**DOI:** 10.1038/srep11975

**Published:** 2015-07-08

**Authors:** Haifeng Qian, Haiping Lu, Haiyan Ding, Michel Lavoie, Yali Li, Weiping Liu, Zhengwei Fu

**Affiliations:** 1 Department of Food Science and Technology, Zhejiang University of Technology, Hangzhou 310032, P. R. of China; 2 College of Agriculture and Biotechnology, Zhejiang University, Hangzhou 310058, P. R. of China; 3 College of Biological and Environmental Engineering, Zhejiang University of Technology, Hangzhou 310032, P. R. of China; 4 Quebec-Ocean and Takuvik Joint International Research Unit, Université Laval, Québec, Canada; 5 College of Environmental and Resource Sciences, Zhejiang University, Hangzhou 310058, P. R. of China

## Abstract

Imazethapyr (IM) is a widely used chiral herbicide that inhibits the synthesis of branched-chain amino acids (BCAAs). IM is thought to exert its toxic effects on amino acid synthesis mainly through inhibition of acetolactate synthase activity, but little is known about the potential effects of IM on other key biochemical pathways. Here, we exposed the model plant *Arabidospsis thaliana* to trace *S*- and *R*-IM enantiomer concentrations and examined IM toxicity effects on the root proteome using iTRAQ. Conventional analyses of root carbohydrates, organic acids, and enzyme activities were also performed. We discovered several previously unknown key biochemical pathways targeted by IM in *Arabidospsis*. 1,322 and 987 proteins were differentially expressed in response to *R*- and *S*-IM treatments, respectively. Bioinformatics and physiological analyses suggested that IM reduced the BCAA tissue content not only by strongly suppressing BCAA synthesis but also by increasing BCAA catabolism. IM also affected sugar and starch metabolism, changed the composition of root cell walls, increased citrate production and exudation, and affected the microbial community structure of the rhizosphere. The present study shed new light on the multiple toxicity mechanisms of a selective herbicide on a model plant.

In recent years the proportion of herbicide consumption over that of all pesticides has increased rapidly, from 20% in 1960 to 48% in 2005^1^. This rapid increase has contributed to agricultural intensification and enhanced crop productivity around the world. However, as a major fraction of applied herbicides leach into soils and groundwater near field crops, intensive pesticide application has also contaminated nearby agricultural areas, exerting toxic effects in non-targeted plants.

Imazethapyr (IM) is one commonly applied chiral herbicide in rice, soybeans, groundnuts and other crops that selectively control dicot weeds^2^. The toxic effect of IM stems from its capacity to strongly inhibit the synthesis of branched chain amino acids (BCAAs: valine, leucine, and isoleucine) in IM-sensitive dicot weeds. It is thought that the primary action mechanism of IM is to inhibit the plant enzyme acetohydroxyacid synthase (E.C.4.1.3.18) or acetolactate synthase (ALS), which catalyzes the first reaction in the biosynthetic pathway of BCAAs^3^. However, recent studies have revealed several other toxicity mechanisms of IM besides inhibition of ALS enzyme activity in monocot or dicot plants, with the *R-*IM enantiomer generally being much more toxic than *S*-IM^4^,^5^. For instance, Zhou *et al.* demonstrated that IM enantiomers selectively damaged root hair growth and significantly reduced the sloughing of border cells from root tips in maize (*Zea mays* L.)^6^. Furthermore, exposure of the rice (*Oryza sativa*) and the model plant *Arabidopsis thaliana* to trace IM concentrations also caused an imbalance in the antioxidant system and disturbed starch and sugar metabolism in an enantioselective manner^5^,^7^.

Even though our knowledge of the IM toxicity effects and mechanisms in plants has improved in recent years, the precise cell mechanisms by which IM enantioselectively exerts its toxic effects are yet to be fully understood. Limited information is available on how IM induces changes in the expression of the whole proteome, and on its toxic effects on a wide range of biochemical pathways in a given plant organism. In order to refine our knowledge of IM toxicity mechanisms, we decided to apply a novel high-throughput technology, i.e. mass spectrometry coupled to protein labeling by isobaric tags for the relative and absolute quantitation (iTRAQ), and analyze the proteome of the roots of a model plant species, *A. thaliana*, in response to a 4-day exposure to 20 μg L^−1^ *S*-IM or *R*-IM. This approach allows the simultaneous identification and quantitative comparison of peptides by measuring peak intensities of reporter ions in tandem mass spectrometry (MS/MS) spectra. With this technology, we have quantitatively identified, to the best of our knowledge, the most complete proteome in *Arabidopsis* roots when compared to other studies^8^,^9^,^10^, i.e. 6,135 proteins, nearly 4000 of which were found to be responsive to *R-* or *S*-IM. Bioinformatics analysis showed that the quantifiable proteins were involved in amino acid synthesis and metabolism, the phenylpropanoid pathway, oxidative stress protection, the TCA cycle, as well as sucrose and starch metabolism. Besides iTRAQ analyses, we also evaluated the effect of IM on the rhizosphere microbial community structure, root enzyme activities as well as on the root concentrations of metals, nutrients, carbohydrate and organic acids (OA) in order to better understand IM toxicity mechanisms. The present study unravels several of the complex molecular bases underlying the enantioselective toxic effects of the herbicide IM in a model plant. This new knowledge could be helpful for genetic engineering of herbicide-resistant crops and management of pesticide application in crops.

## Results

### The enantioselective effects of IM on roots, shoots and leaves size and morphology

**Figure 1:**
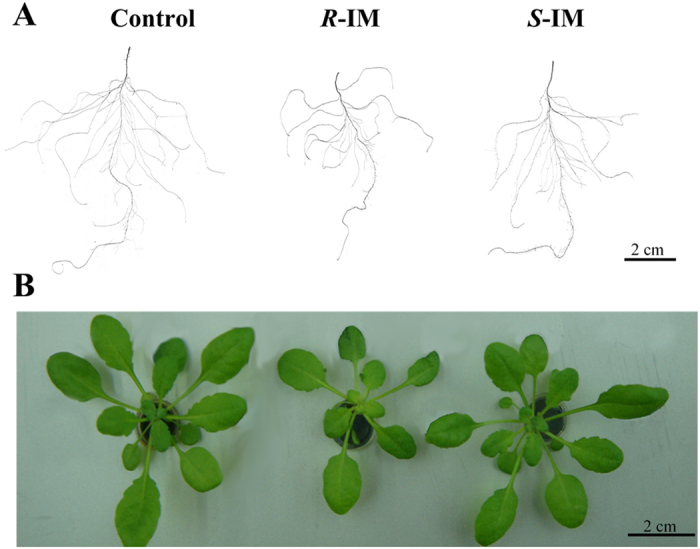
Photos of IM enantiomer-treated *A. thaliana.* The phenotype of roots (**A**) and seedlings (**B**) after a 4-day exposure to 20 μg L^−1^ *R*-IM or *S*-IM compared with those in control conditions.

Sizes of roots, shoots and leaves all decreased in response to the 4-day exposure to 20 μg L^−1^*S*-IM an *R*-IM (Fig. 1). Exposure to IM significantly decreased shoot and roots fresh weight, total root length, root surface area, and number of root tips, but significantly increased root diameter (Table 1). For all toxicity endpoints, the inhibitory effect of the *R*-IM enantiomer on root elongation was significantly greater than that of the *S*-IM enantiomer (Table 1).

### The enantioselective effects of IM on root elemental absorption

Both IM enantiomers significantly increased Cu accumulation and significantly decreased Mg, Na and Zn accumulation, but did not alter the accumulation of Ca, P and Mn. *R*-IM generally showed stronger stimulatory and inhibitory effects on elemental absorption than *S*-IM, except for K and Fe accumulation for which only the *S*-IM enantiomer has a stimulatory effect (Table 2).

### Identification and relative quantitation of the *Arabidopsis* root proteome using iTRAQ

**Figure 2:**
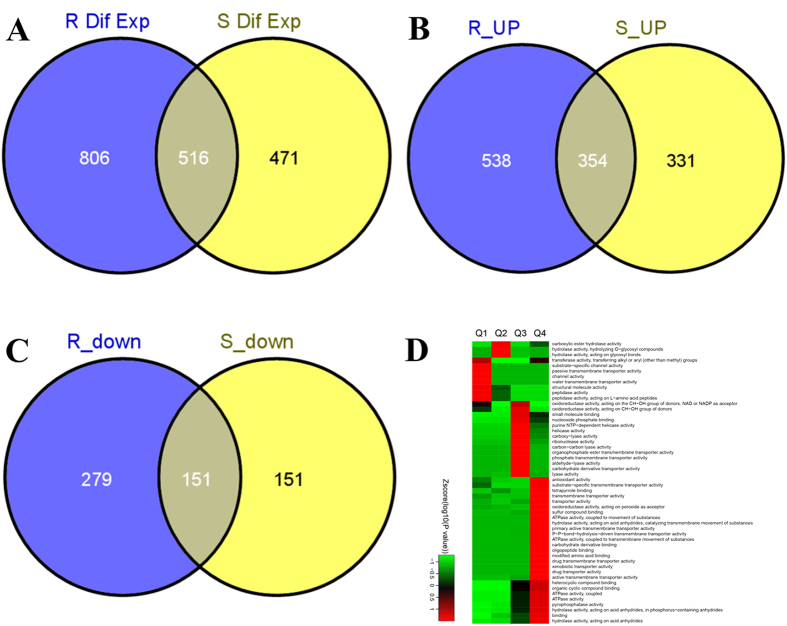
Statistical analysis of the proteins detected in iTRAQ experiment. Numbers of differentially expressed proteins in *A. thaliana* roots (**A**) upregulated proteins in *Arabidopsis* roots (**B**) and downregulated proteins in *Arabidopsis* roots (**C**) Gene ontology for molecule functional classification after *R*-IM treatment. The ratios between the protein content in *R*-IM (or *S*-IM) exposed plants and that in the control group were used to express the level of up- or down-regulation in protein expression. These ratios were classified in four groups (Q1 to Q4); two groups for down-regulation (Q1: ratio = 1/1.5; Q2: 1/1.5 < ratio < 1/1.3) and two groups for up-regulation (Q3: 1.3 < ratio < 1.5, Q4: ratio > 1.5). The color index (Zscore) is showed in the legend. According to the Zscore, the red color represents the proteins that were significantly accumulated in a molecular function GO (Gene ontology) term. The green color shows that the proteins were not significantly related with a given molecular function (**D**).

In the 3-plex iTRAQ-based quantitative proteomic studies, 6,135 proteins were identified in *A. thaliana* roots. To our knowledge, this study represents the most comprehensive proteomic analysis of *A. thaliana* roots to date^8^,^9^,^10^. Among these 6,135 root proteins, 4,185 and 3,802 proteins were quantified in *R*- and *S*-IM treatments, respectively. 1,322 and 987 proteins were differentially expressed upon treatment with *R*- and *S*-IM, respectively (Fig. 2A). Among these differentially expressed proteins, 892 and 685 proteins were up-regulated by *R*- and *S*-IM treatment, respectively (Fig. 2B) (the ratio between IM treatment and control was >1.3) and 354 of the up-regulated proteins overlapped between both treatment groups. In addition, 430 and 302 proteins were down-regulated by *R*- and *S*-IM treatments, respectively (Fig. 2C) (the ratio between IM treatment and control was <1/1.3). Of the two groups of down-regulated proteins, 151 proteins overlapped. Bioinformatics analysis showed that the quantifiable proteins were involved in several cellular functions including amino acid synthesis and metabolism, the phenylpropanoid pathway, sucrose and starch metabolism, and the TCA cycle (Fig. 2D).

### The enantioselective effects of IM on amino acid synthesis and catabolism

Because IM is thought to control weeds primarily by reducing the levels of three BCAAs (isoleucine, leucine and valine) through the inhibition of acetolactate synthase (ALS), we first analyzed the proteins associated with amino acid metabolism (Table S1). Surprisingly, neither *R*- nor *S*-IM exposure inhibited the expression of the small ALS subunit (AT2G31810.1), but *R*-IM increased the abundance of the large ALS subunit (AT3G48560.1) to 1.3-fold that of the control.

Even though IM did not affect the expression of the ALS enzyme, our analysis of the proteomic response of *A. thaliana* to IM showed that this herbicide decreased the expression of a number of proteins involved in BCAA synthesis (Table S1). Indeed, *R*-IM decreased isopropylmalate isomerase 1 (AT3G58990.1) and 3-isopropylmalate dehydrogenase 1 (AT1G31180.1) expression to 1.5- and 2.0-fold that of the control, thereby inhibiting Leu and Ile synthesis. Moreover, *S*-IM decreased 3-isopropylmalate dehydrogenase 2 (AT1G80560.1) to 1.3-fold that of the control, but it increased ketol-acid reductoisomerase (AT3G58610.3) and BCAA aminotransferase 4 (AT3G19710.1; involved in leucine and isoleucine biosynthesis^11^) to 1.3- and 1.5-fold that of the control.

Furthermore, our proteomic analysis showed that IM stimulated BCAA catabolism (Table S1). Indeed, IM increased the expression of a number of enzymes involved in BCAA catabolism. As shown in Table S1, Acyl-CoA dehydrogenase-related protein (AT3G06810.1), methylcrotonoyl-CoA carboxylase subunit alpha (AT1G03090.2) and beta chain (AT4G34030.1), hydroxymethylglutaryl-CoA lyase (AT2G26800.1), and aldehyde dehydrogenase (AT1G54100.2) were all up-regulated by *R*-IM, but the *S*-IM enantiomer only increased the expression of branched-chain aminotransferase 4 (AT3G19710.1). Again, the *R*-IM enantiomer was more toxic than the *S*-IM enantiomer as demonstrated in the proteome of *A. thaliana*.

### The enantioselective effects of IM on sucrose and starch metabolism

**Figure 3:**
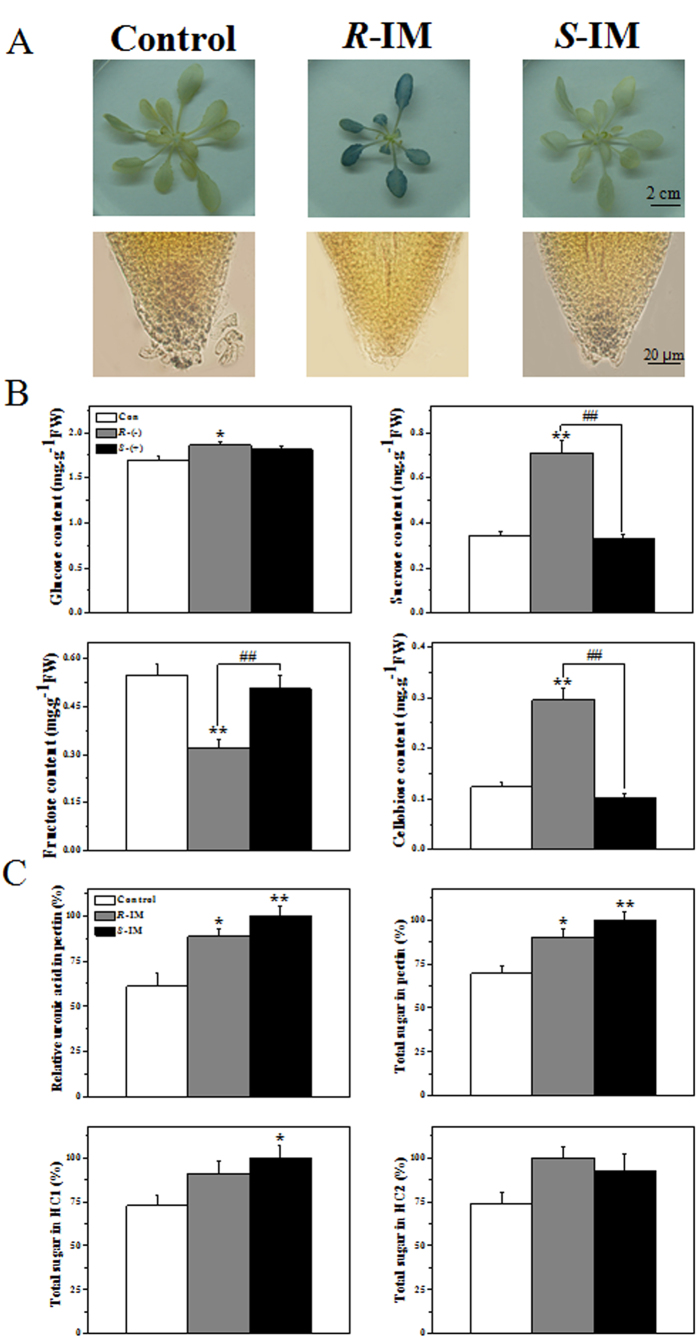
Carbohydrate and starch content in *A. thaliana* roots. Starch content in plant shoot and leaves (first row) and plant root tips (second row) stained with KI-I after IM-enantiomer exposure (**A**) The glucose, sucrose, fructose and cellobiose contents (**B**) Cell wall polysaccharide content (**C**) The * and ** indicate that the values are significantly different from those of the control plants at p < 0.05 and p < 0.01, respectively. ##indicates that the numbers are significantly different from those of *S*-IM-exposed plants at p < 0.01. Error bars are standard errors of four biological replicates.

The expression of several sucrose- and starch-responsive proteins involved in carbohydrate metabolism was affected by IM (Table S1). Sucrose synthesis catalyzed by sucrose synthase (SUS) is widely believed to be the primary route of entry of carbon into cellular metabolism in plants^11^. The abundances of SUS1 (AT5G20830.2), SUS4 (AT3G43190.1), SUS5 (AT5G37180.1) and SUS6 (AT1G73370.1) were increased by treatment with each enantiomer, *R*- and *S*-IM. Among these SUS proteins, SUS4 expression in the root is higher than in other *A. thaliana* tissues according to Bieniawska *et al.*^12^. The results of KI-I staining indicated that the starch level decreased after IM treatment, especially after *R*-IM treatment (Fig. 3A).

### The enantioselective effects of IM on cell wall composition

Exposure to the *R*- or *S*-IM enantiomer increased the glucose, sucrose and cellobiose contents, which are key biomolecules involved in cell wall synthesis; with the *R*-IM enantiomer having a greater stimulatory effect than the *S*-IM enantiomer (Fig. 3B). Increases in the expression levels of basic structural units of plant cell walls, i.e. polysaccharides such as pectin, hemicellulose 1 (HC1) and hemicellulose 2 (HC2) further confirmed that cell wall biosynthesis was enhanced (Fig. 3C).

### The enantioselective effects of IM on phenylpropanoid biosynthesis

iTRAQ analyses showed that *R*-IM, but not *S*-IM, increased the expression of several phenylalanine ammonia lyase (PAL) (Table S1). More specifically, PAL1 (AT2G37040.1), PAL2 (AT3G53260.1), PAL4 (AT3G10340.1), these enzymes catalyzed the biosynthesis of phenylpropanoids, a group of phenylalanine-derived physiologically active secondary metabolites such as lignins, flavonols, isoflavonoids and anthocyanins^13^,^14^. For instance, our previous report demonstrated that *R*-IM induced the biosynthesis of anthocyanins^15^, which comprised antioxidants that protect plants against biotic and abiotic stressors and decrease reactive oxygen species (ROS) accumulation and toxicity^16^.

### The enantioselective effects of IM on the synthesis of proteins and organic acids involved in the tricarboxylic acid cycle

**Figure 4:**
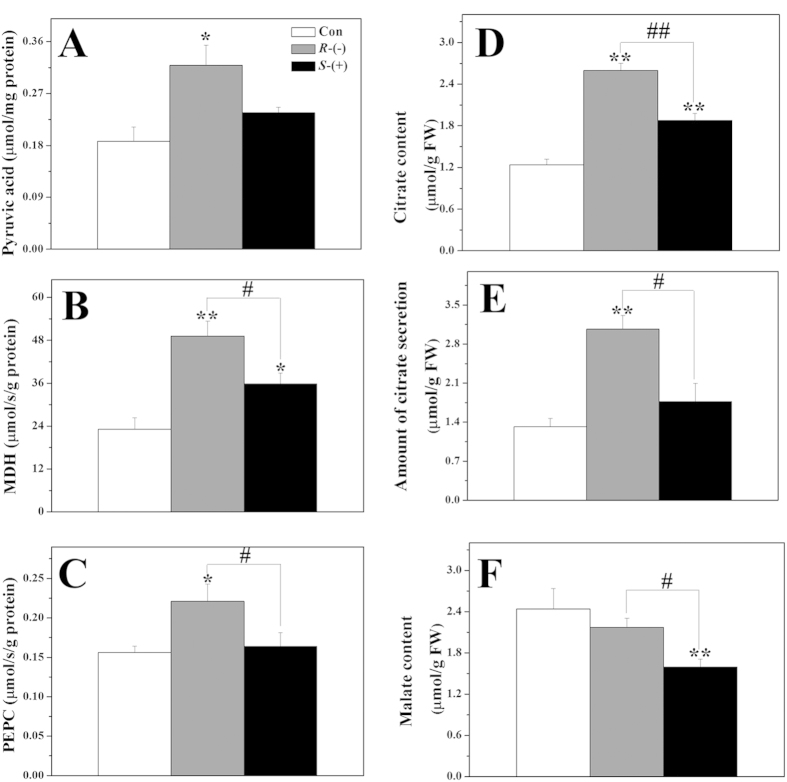
The organic acid levels and enzyme activities in the TCA cycle after IM enantiomer exposure. Pyruvate content (**A**) malate content (**B**) citrate content (**C**) amount of citrate exuded in the external medium (**D**) Activity of PEPC (**E**) and MDH (**F**). * and ** indicate that the values are significantly different from those of the control plants at p < 0.05 and p < 0.01, respectively. # and ## indicate that the numbers are significantly different from those of the *S*-IM-exposed plants at p < 0.05 and p < 0.01, respectively. Error bars are standard errors of four biological replicates.

The mean pyruvate concentration increased to 1.7-fold that of the control after treatment with *R*-IM (Fig. 4A). iTRAQ analyses revealed that *R*-IM treatment greatly increased the expression of several TCA-cycle enzymes, e.g., isocitrate dehydrogenase (ICDH, AT1G54340.1), 2-oxoglutarate dehydrogenase (AT5G65750.1), succinyl-CoA ligase [ADP-forming] subunit alpha-1(AT5G08300.1), and malate dehydrogenase (MDH, AT5G56720.1) (Table S1). Moreover, *R*-IM repressed the abundance of ATP-citrate lyase A-1 (ACLA-1, AT1G10670.4), which is a key enzyme producing acetyl-CoA from citrate during fatty acid synthesis in the chloroplast. In contrast, *S*-IM increased the expression of ATP-citrate lyase A-3 (ACLA-3, AT1G09430.1).

As observed for *R*-IM treatment, *S*-IM treatment also increased the abundance of several TCA-related proteins, such as citrate synthase 2 (CS, AT3G58750.1), the 2-oxoglutarate dehydrogenase E1 component (AT3G55410.1) and MDH leading to an increase in oxaloacetate (OA) synthesis. The activities of OA synthesis-related enzymes were also stimulated in response to *S*-IM (MDH) and *R*-IM treatment (MDH and PEPC) (Fig. 4B,C). Furthermore, the *S*-IM-induced decrease in the expression of succinate dehydrogenase iron-sulfur subunit 1 (AT3G27380.2) should also contribute to a reduction in respiration rate through the TCA cycle compared to that in the presence of *R*-IM.

In total, approximately 10 proteins encoded by the mitochondrial genome were induced by *R*-IM, which should stimulate respiration through the TCA cycle and cause OA synthesis (Table S1). Overall, the protein expression and enzymatic analysis are consistent with an increase in citrate concentrations in response to *S*-IM and *R*-IM exposure as measured by HPLC; *R*- and *S*-IM exposure significantly increased citrate synthesis by 2.1- and 1.5-fold that of the control, respectively (Fig. 4C) and *R*-IM treatment also noticeably promoted citrate exudation (Fig. 4D).

### The enantioselective effects of IM on microbial community structures in the root

**Figure 5:**
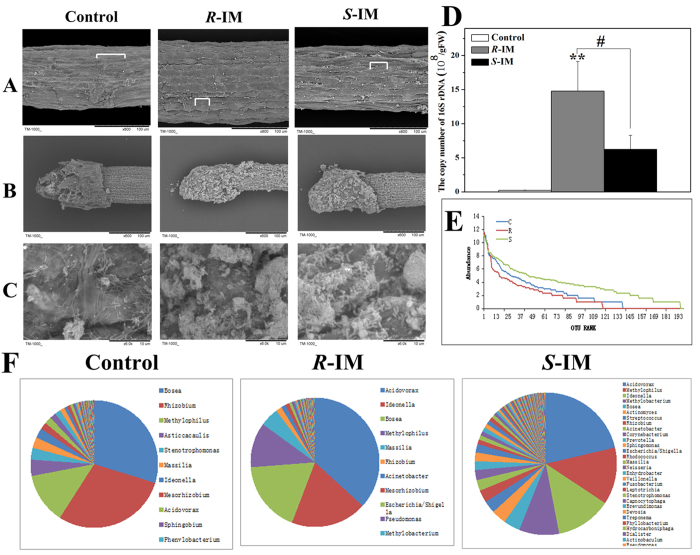
Scanning electron micrographs of the root surface and microbial community structure analysis. (**A**) Root surface in mature region, (**B**) Root tissue, (**C**) Microorganism film in root tissue (A, magnification = 800×; B, magnification = 600×; C, magnification = 6000×). (**D**) The rank abundance curve, (**E**) The microorganism species distribution of each sample after IM-enantiomer treatment. ** indicates that the values are significantly different from those of the control plants at p < 0.01. # indicates that the values are significantly different from those of *S*-IM-exposed plants at p < 0.05. Error bars are standard errors of four biological replicates.

We observed the root structure of IM-treated plants (grown in non-sterile conditions) by scanning electron microscopy (SEM). Root cell length decreased and its diameter increased in response to IM exposure, especially after *R*-IM treatment (Fig. 5A). We also observed bacterial biofilms on the root tips of *A. thaliana* exposed or not to IM, and the quantity of bacterial biofilms appears to be higher for the plants exposed to *R*-IM and *S*-IM compared to the control (Fig. 5B,C). We subsequently estimated the abundance of bacteria in the root tips of each treatment by amplifying the 16s rDNA genes (specific to bacteria) by qRT-PCR. Our results confirmed that the bacterial abundance in the rhizosphere increased in response to *R*- or *S*-IM treatment compared to the control. We found 1.5 × 10^9^ copies of 16S rDNA/mg root F.W. (fresh weight) in the *R*-IM-treated sample and 6.2 × 10^8^ copies of 16S rDNA/mg F.W. in the S-IM treated sample whereas only 2.3 × 10^7^ copies of 16S rDNA/mg F.W. were measured in the control (Fig. 5D).

To obtain a snapshot of the microbial community structure in the biofilm colonizing the root tips of plants exposed or unexposed to IM, we then performed 454-pyrosequencing of the 16S rRNA genes found in the bacterial biofilm sampled around the roots of our xenic *A. thaliana* cultures. The bacterial diversity was similar for all definitions of OTU^17^ (Operational taxonomic unit, an operational definition of a species or group of species often used when only DNA sequence data are available) among the *R*-IM, *S*-IM and control samples (Table 3). The Chao and ACE indices of species richness^18^ were highest in the *R*-IM treatment and was higher in the *S*-IM treatment than in the control), reaching average values at the unique sequence level of 385.44/485.71 (Chao/ACE index) for *R*-IM treatment, 304/304.53 (Chao/ACE index) for *S*-IM treatment and 257.03/261.54 for the control. The *S*-IM-treated group appeared to be more diverse based on two widely used diversity indices, the Shannon’s^19^ and Simpson’s indices^20^ of diversity. In addition, the *R*-IM-treated group showed the lowest diversity among the three groups based on the coverage index (Table 3). In line with the diversity index results, the homogeneity of the distribution of the microbial community species in the rhizosphere based on the rank abundance curve was lower in the *R*-IM treatment compared to the *S*-IM treatment and control (Fig. 3E). The proportion of *Acidovorax* out of all species in the biofilm reached 36.8% and 21.4% in the *R*- and *S*-IM groups, respectively, but was only 2% in the control (Fig. 3F).

## Discussion

Trace IM concentrations markedly affected the growth of the model plant *A. thaliana* (Fig. 1, Table 1); the *R*-IM enantiomer being more toxic on root, shoot and leaves growth than the S-IM enantiomer as reported in previous studies^5^,^15^. The root volume was significantly reduced by *R*-IM treatment, but it was not greatly affected by *S*-IM. Interestingly, the root diameter was significantly increased by IM treatments, especially in the presence of *R*-IM, which may cause enlarged cortical cells, pith extension, and stelar aberrations in the primary root^21^.

iTRAQ analyses and other conventional physiological/biochemical/chemical measurements suggest that the strong toxic IM effect on *A. thaliana* stems from IM-induced perturbations of multiple biochemical pathways and cellular functions; the *R*-IM enantiomer being consistently more toxic than the *S*-IM enantiomer. IM exposures were shown to perturb photosynthesis, amino acid synthesis and metabolism, phenylpropanoid pathway, sucrose and starch metabolism, and the TCA cycle (Fig. 2D).

The elemental analysis of *A. thaliana* roots provided insights into the toxicity mechanisms of IM on the physiology of photosynthesis. Since Mg is necessary for chlorophyll synthesis, IM-induced Mg deficits in roots in the presence of *S*-IM and particularly *R*-IM could contribute to the reported decrease in chlorophyll synthesis and photosynthesis in *Arabidopsis* exposed to 2.5 μg L^−1^ IM for 2–4 weeks^15^.The IM-induced over-absorption of Cu could promote excessive production of reactive oxygen species (ROS), damage chloroplast membranes, and impair chlorophyll synthesis as shown in algae exposed to Cu in the studies of Wei *et al.* and Ouzounidou^22^,^23^. The previously reported toxic effect of trace *S*-and, particularly *R*-IM concentrations on the chloroplast and photosynthesis of *Arabidopsis* as well as the IM-induced ROS production in *Arabidopsis* may well be linked at least partly to perturbation in Cu and Mg accumulation^5^,^15^.

One of the most important toxicity targets of IM is thought to be the ALS enzyme involved in BCAA synthesis^3^,^24^. Since our results show that the expression of this enzyme remains unaffected by IM, we conclude that IM decreases BCAA synthesis by inhibiting the activity of the ALS enzyme, but does not affect the gene transcription or translation of the ALS enzyme. In contrast, IM induced a decrease in the expression of five proteins involved in BCAA synthesis (Table S1); this may be another reason why the BCAA content in plants decreased after IM treatment, especially after *R*-IM treatment^25^. iTRAQ proteomic analyses further suggest that the toxic effect of IM on BCAA synthesis is not only related to its inhibitory effect on BCAA gross synthesis as classically thought, but that IM also increased the catabolism of BCAA by increasing the expression of six different enzymes involved in BCAA catabolism^11^. A possible biochemical pathway by which BCAAs are catabolized would be the BCAAs conversion into acyl-CoA derivatives in the mitochondrion, which are converted through subsequent reactions into either acetyl-CoA or succinyl-CoA and then enter the TCA cycle^13^,^14^.

To better understand the cellular mechanisms related to the above mentioned IM-induced decrease in amino acid synthesis, we studied several proteins and organic acid of the TCA cycle. We found that *R*-IM exposure significantly repressed the expression of ATP-citrate lyase (ACLA) (Table S1). ACLA suppression was also shown to affect plant phenotypes, inhibiting primary root elongation^26^,^27^, as observed in Fig. 1. This inhibition in ACLA activity should decrease fatty acid synthesis in the chloroplast and increase acetyl-CoA concentration in the mitochondrion and thus stimulate the TCA cycle. The IM-induced inhibition of the ALS activity and proteins involved in BCAA synthesis as well as its increase of BCAA catabolism (Table S1) should also increase mitochondrial acetyl-CoA, pyruvate, citrate concentrations and up-regulate the TCA cycle. We indeed found that the expression of several proteins of the TCA cycle were upregulated in IM-treated plants (iTRAQ results, Table S1). In response to IM-induced TCA cycle up-regulation, we also measured a 1.7-fold increase in pyruvate concentrations in response to *R*-IM exposure (Fig. 4A). An increase in pyruvate concentration has also been previously reported in corn and peas exposed to other ALS-inhibiting herbicides^28^,^29^. Even though the malate root content significantly decreased in response to IM exposure (Fig. 4F), the root citrate concentrations and citrate exudation increased in IM-treated plants (Fig. 4C,D). The observed increase in root intracellular citrate concentrations could up-regulated the expression of alternative oxidase. This enzyme is involved in mitochondrial retrograde signaling, which affect multiple biochemical pathways to cope with a cell stressor^27^,^30^,^31^. In the present study, we indeed observed an increase in alternative oxidase expression (4.4- and 1.7-fold of the control with *R*- and *S*-IM treatment, respectively, Table S1), implicating that IM caused TCA cycle dysfunctions and mitochondrial retrograde signaling, which may affect other metabolic pathways, such as sugar and starch metabolism, and phenylpropanoid biosynthesis, as discussed below.

IM also affected starch synthesis through the inhibition of several sucrose synthase enzymes (SUS1, SUS4, SUS5 and SUS6). The SUSs are required for defense against biotic and non-biotic stresses, and are thus strongly affected by pathogen interactions and anoxia in several species^32^,^33^,^34^. An increase in SUS activity favored the generation of UDP-glucose, which is directly used for starch, cell wall, and callose synthesis^35^,^36^. As KI-I coloration (Fig. 3A) shows that starch production decreased after IM treatment, especially after R-IM treatment, but cell wall polysaccharides increased upon IM exposure (Fig. 3C), it is strongly suggested that the IM-induced up-regulation of sucrose synthesis is invested in cell wall biosynthesis rather than starch production. Note also that the IM-induced up-regulation of phenylpropanoid synthesis (iTRAQ results in Table S1) such as lignins, an important component of cell wall, is in line with the above mentioned effects of IM on cell wall biosynthesis. Two hypotheses might explain the up-regulation in cell wall biosynthesis induced by IM. First, the enhancement in cell wall biosynthesis might be a toxic effect of IM. Second, up-regulation of cell wall biosynthesis might be triggered to repair the cell wall damages caused by IM. Note that a potential increase in the amount of negatively charged polysaccharides in the cell wall should not sensitively decrease the uptake of the anionic IM species at pH higher than the pKa, because the cell permeability of the anionic species of herbicides is typically 1 000 to 10 000 times lower than the permeability of the neutral protonated species. Generally, only the neutral protonated species of weak acid herbicides is thought to be appreciably accumulated in plants at pH higher than the pKa^37^.

**Figure 6:**
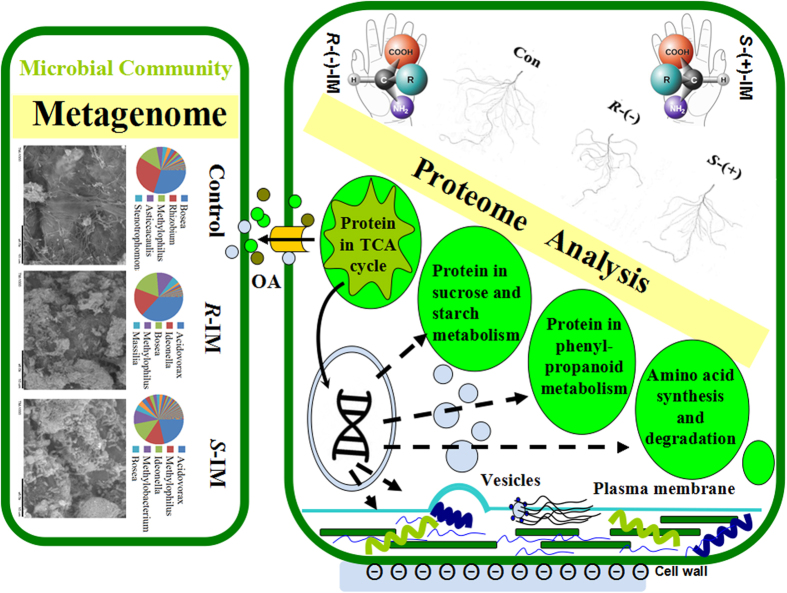
Conceptual scheme describing the toxic effects of IM in *A. thaliana* roots and the potential influence of organic acid secretion on the rhizosphere bacterial community.

Our results not only provide a comprehensive analysis of IM toxicity effects in *A. thaliana*, but also provide evidence that the toxic effect of IM on *A. thaliana* is related to changes in diversity and abundance of microorganisms in the rhizosphere (See Fig. 6 for a conceptual scheme summarizing the multiple IM toxic effects in *A. thaliana*). Roots are known to excrete secondary metabolites that act as messengers that attract *Rhizobium* and *arbuscular mycorrhizal* fungi^38^. More specifically, Rudrappa *et al.* demonstrated that the secretion of the TCA cycle intermediate L-malic acid from *A. thaliana* roots is used to recruit the beneficial rhizobacterium *Bacillus subtilis*^39^. Therefore, in the present study, the measured increase in citrate exudation from *A. thaliana* roots (Fig. 4D) in response to IM stress could explain the observed increase in *B. subtilis* abundance in the rhizopshere of IM-treated *A. thaliana*. Furthermore, the increase in *Acidovorax*, an acidophilic bacterium^40^, abundance in the rhizosphere (Fig. 3F) of IM-treated plants could also be related to exudation of organic acid from plant roots^41^,^42^. Further studies are clearly needed to confirm/infirm the above hypothetical interactions between bacterial community structure of the rhizosphere and IM.

The changes in bacterial abundance and community structure in the rhizosphere in response to IM exposure could also be affected by IM-induced differences in root morphology (root ramifications, diameter, volume) and/or direct toxic effect of IM on bacteria in the rhizosphere. Irrespective of the precise mechanism(s) explaining the effect of IM on the bacterial community structure of the rhizosphere, the strong effect of IM on the microorganisms of the rhizosphere revealed in this study is important because it is expected to affect plant nutrition and perhaps indirectly modulate IM toxicity in *A. thaliana*.

## Methods

### Plant culture and IM exposure conditions

*A. thaliana* ecotype Columbia seeds were sterilized with ethanol (75%) and HgCl (0.1%). The sterile seeds were subsequently vernalized at 4 °C for 4 days and transferred onto sponges, which were placed into 1.5 mL Eppendorf tubes from which the bottoms were cut, allowing root growth into the nutrient solution. Murashige and Skoog (MS) nutrient solution was used (composition described in Zhu *et al.*) and the intial pH was adjusted to 5.6^43^. After 4 weeks under hydroponic conditions, the uniform seedlings were exposed for 4 d to 5,10, 20, and 40 μg L^−1^*R*- or *S*-IM in MS nutrient solution (initial pH = 5.6). The plants were grown in a non-sterile growth chamber at 25 °C under a 12 h light/12 h dark cycle at a light intensity of 40 μmol photons m^−2^ sec^−1^. The concentration of 20 μg L^−1^ IM, which was close to the IC50 on the root fresh weight of *A. thaliana* (Table 1), was chosen for all the proteomics and biochemical analysis performed in the present study. Note that chiral separation of enantiomers was performed on a Jasco LC-2000 series HPLC system (Jasco, Tokyo, Japan) equipped with an OJ chiral column (4.6 mm I.D. × 250 mm, Daicel chemical industries, Japan), as reported in Qian *et al.*^4^. Note also that IM is an amphoteric compound with acidic ionizable carboxyl groups (pKa1 = 3.9) and basic amine functional groups (pKa2 = 2.1)^44^. Therefore, at pHs > pKa1 (as expected in our IM exposure experiments) the anionic species IM^−^ predominates.

### Measurements of root size and morphological parameters

After 4 d of exposure, an automatic root-scanning apparatus (MINMAC, STD1600+) equipped with WinRHIZO software (Regent Instruments Corporation) was used to measure several root morphological parameters for each plant, i.e. total root length, surface area, volume, diameter, and number of root tips. Four replicates were used in each treatment, and each replicate consisted of 5 randomly selected seedlings.

### Analyses of nutritional elements, carbohydrates, polysaccharides, starch and uronic acid in plant roots

To measure the concentrations of nutritional metals (Cu, Fe, Mn, Zn) and other nutritional elements (Ca, Mg, Na, P) in roots of *A. thaliana*, control and IM-treated roots of *A. thaliana* were collected, dried, transferred in acid-washed plastic containers and digested in the microwave with 68% HNO_3_ for 5 min at 700 W, followed by 10 min at 700–1200 W and 20 min at 1200 W. Subsequently, samples for metal analyses were diluted in Milli-Q water to achieve a final 5% v/v HNO_3_ concentration prior to analysis by inductively-coupled plasma mass spectrometry (ICP-OES, PerkinElmer Optima 4300DV).

The content of glucose, sucrose, and fructose in the roots of *A. thaliana* was measured according to the procedure detailed in Qian *et al.*^5^. The content of polysaccharides and uronic acid in plant cell walls was measured according to the method published by Zhu *et al.*^44^. The starch content in the whole plants or roots was evaluated semi-quantitatively by KI-I staining. The whole plant was transferred to 80% ethanol, pooled the ethanol after incubated in a boiling-water bath for 3 min, and added the new ethanol (repeated three times) to remove pigments, and then stained for 5 min with KI-I^5^. The root tissues were stained according to the method of Takahashi *et al.*^45^.

### Estimation of 16S rDNA abundance in the rhizosphere by qRT-PCR

We quantified the 16S rDNA abundance per root fresh weight by qRT-PCR according to the method detailed in Qian *et al.*^46^.

### 454-pyrosequencing of the 16S rRNA gene

Metagenomic sequencing was performed according to Bowman *et al.*^47^.The 4-d-treated roots were washed with MS liquid medium without herbicides, and their DNA was extracted by the phenol-chloroform method. A section of the 16S rRNA gene (V3-V5) was amplified, and sequencing was performed on a GS FLX pyrosequencer (Roche 454 GS FLX+).

### Total protein extraction and LC-MS/MS analysis

After treatment, crude proteins from *A. thaliana* roots (approximately 0.4 g fresh weight) were extracted and digested with trypsin (Promega) at an enzyme-to-substrate ratio of 1:50 for 12 h at 37 °C. After iTRAQ (AB Science) labeling, equal amounts of labeled peptides from each group were mixed and resolved into 15 fractions by high performance liquid chromatography (HPLC), followed by Q Exactive mass spectrometry (Thermo Fisher Scientific). The resulting MS/MS data were searched against a Uniprot *Arabidopsis* protein database with MaxQuant (v1.0.13.13) with 1% FDR (False discovery rate). A functional annotation of the quantified proteins was performed with GO (Gene ontology), KEGG (Kyoto encyclopedia of genes and genomes) pathway analysis, functional enrichment analysis and secondary structure analysis. Two technical replicates with tag swapping were also conducted for each biological replicate. Further details on MS/MS raw data database searching, peptide and protein identification and quantification are provided in supplemental materials (Table S1).

### Data treatment and statistical analyses

The iTRAQ results for protein identification and quantitation were selectively filtered before exportation according to several criteria. An ion score or expected cutoff less than 0.05 (with 95% confidence) was required for protein identification. Two criteria were used for the quantitation of the identified proteins: 1) the median protein ratio was chosen (excluding the outliers); 2) the minimum precursor charge was set to 2 and only unique peptides were used for quantitation. A 1.3-fold up- or down-regulation was chosen to identify significant over- or under-expression, respectively, of proteins in addition to P-value < 0.05.

Statistical differences among the mean root contents in polysaccharides, metals, organic acids as well as in root enzyme activity (p < 0.05) were tested with analyses of variance (ANOVA) using the program StatView. The preliminary conditions of ANOVAs (normality and homogeneity of variances of residuals) were tested and if the conditions were violated, the data were transformed prior to ANOVAs.

## Additional Information

**How to cite this article**: Qian, H. *et al.* Analyzing *Arabidopsis thaliana* root proteome provides insights into the molecular bases of enantioselective imazethapyr toxicity. *Sci. Rep.* **5**, 11975; doi: 10.1038/srep11975 (2015).

## Supplementary Material

Supplementary Information

## Figures and Tables

**Table 1:** Root size (length, surface, diameter, volume), number of root tips as well as fresh weight (FW) of roots and shoots in *Arabidopsis thaliana* exposed for 4 d to 20 μg L^−1^*S*-IM and *R*-IM enantiomers compared with those in control conditions.

	**Shoot FW (mg plant**^**−1**^)	**Root FW (mg plant**^**−1**^)	**Total RL (cm plant**^**−1**^)	**Root SA (cm**^**2**^**plant**^**−1**^)	**Root D (mm)**	**Root V (cm**^**3**^**plant**^**−1**^)	**Number of tips (plant**^**−1**^)
Control	100.10 ± 2.24	11.50 ± 0.21	207.98 ± 7.97	14.75 ± 0.69	0.23 ± 0.00	0.08 ± 0.01	872.40 ± 40.14
*R*-IM	68.05 ± 1.93**##	5.00 ± 0.14**##	124.97 ± 4.42**##	10.51 ± 0.34**##	0.27 ± 0.01**##	0.07 ± 0.00*#	448.67 ± 18.72**##
*S*-IM	89.30 ± 1.94**	6.50 ± 0.35**	168.19 ± 5.42**	13.22 ± 0.40*	0.25 ± 0.01**	0.08 ± 0.00	759.73 ± 22.83**

FW: fresh weight; RL: root length; SA: surface area; D: diameter; V: volume. *Represents a statistically significant difference when compared with the control (*p < 0.05; **p < 0.01). #Represents a statistically significant difference when compared with *S*-IM-exposed plants (#p < 0.05; ##p < 0.01). Mean of four biological replicates ± standard error (S. E.).

**Table 2:** The effect of a 4-d exposure of *Arabidopsis thaliana* to 20 μg L ^−1^*S*-IM and *R*-IM on the root concentrations of several elements (in μg/g dry weight or DW).

**element**	**Control (μg/g DW)**	**R-IM (μg/g DW)**	**S-IM (μg/g DW)**
K	89369.92 ± 2377.43	89336.32 ± 1378.66#	96821.96 ± 2424.42*
Mg	1975.33 ± 30.40	1141.73 ± 17.61**##	1618.00 ± 71.91**
Ca	6018.93 ± 49.60	6060.10 ± 57.95	6037.21 ± 63.69
Fe	6120.95 ± 121.77	5955.50 ± 14.00##	6502.11 ± 133.11*
Na	655.89 ± 5.41	402.67 ± 7.10**##	486.42 ± 11.04**
P	13087.96 ± 101.89	13456.99 ± 194.01#	12981.71 ± 102.55
Mn	13.94 ± 0.18	13.35 ± 0.20	13.11 ± 0.71
Zn	532.18 ± 11.33	277.17 ± 3.09**##	372.00 ± 8.29**
Cu	21.18 ± 0.19	32.83 ± 0.26**##	25.47 ± 0.29**

*Represents a statistically significant difference when compared with the control (*p < 0.05; **p < 0.01). #Represents a statistically significant difference when compared with S-IM-exposed plants (#p < 0.05; ##p < 0.01). Mean of four replicates ± standard error (S. E.).

**Table 3:** Operational taxonomic unit (OTU) abundance and diversity index (Chao, ACE, Simpson, Shannon, Coverage) in bacterial biofilms sampled in the rhizosphere of *A. thaliana* exposed or not to *R*- or *S*-IM.

**Group**	**OUT**	**Chao**	**ACE**	**Simpson**	**Shannon**	**Coverage**
Control	196	257.03	261.54	0.12	2.87	0.994005
*R*-IM	213	385.44	485.71	0.18	2.41	0.988632
*S*-IM	249	304	304.53	0.07	3.60	0.995237
