# Supplementary material for: Analyzing Arabidopsis thaliana root proteome provides insights into the molecular bases of enantioselective imazethapyr toxicity
Source: Sci Rep. 2015 Jul 8;5:11975. doi: 10.1038/srep11975 (PMC4495388; doi:10.1038/srep11975)
Supplement: Supplementary Information [file srep11975-s1.pdf]

Analyzing *Arabidopsis thaliana* root proteome provides insights into the molecular bases of enantioselective imazethapyr toxicity

Haifeng Qian[1,3], Haiping Lu[2], Haiyan Ding[3], Michel Lavoie[4],Yali Li[3], Weiping Liu[5], Zhengwei Fu[3],*

1 Department of Food Science and Technology, Zhejiang University of Technology, Hangzhou 310032, P. R. of China

2 College of Agriculture and Biotechnology, Zhejiang University, Hangzhou 310058, P. R. of China

3 College of Biological and Environmental Engineering, Zhejiang University of Technology, Hangzhou 310032, P. R. of China

4 Quebec-Ocean and Takuvik Joint International Research Unit, Université Laval, Québec, Canada

5 College of Environmental and Resource Sciences, Zhejiang University, Hangzhou 310058, P. R. of China

***Corresponding author:**

Zhengwei Fu

College of Biological and Environmental Engineering, Zhejiang University of Technology, Hangzhou, 310032, P. R. of China

E-mail: azwfu@zjut.edu.cn

Supporting Information

Table S1. Differentially accumulated proteins and their assigned or assumed functions. Proteins were classified as differentially expressed if the average change in both experiments was greater than 1.30-fold and 0.769-fold for up- and downregulated proteins, respectively.

Protien IPI ID	Protein description	R/C median Ratio	S/C median Ratio	R/S medianRatio
**amino acid synthesis and degradation**				
IPI00545682 (AT3G58990.1)	IPMI1, Isopropylmalate isomerase 1	0.69±0.08	0.83±0.14	0.82±0.05
IPI00544447 (AT1G31180.1)	IMD3, 3-isopropylmalate dehydrogenase 1, chloroplastic	0.50±0.22	_	1.03±0.06
IPI00522721 (AT1G80560.1)	IMD2, 3-isopropylmalate dehydrogenase 2, chloroplastic	0.77±0.05	0.76±0.18	1.06±0.17
IPI00526442 (AT3G19710.1)	BCAT4, Probable branched-chain-amino-acid aminotransferase 4	0.95±0.07	1.52±0.01	0.66±0.04
IPI00533630 (AT3G58610.3)	Ketol-acid reductoisomerase, chloroplastic	1.04±0.16	1.35±0.21	0.76±0.10
IPI00529405 (AT3G06810.1)	IBR3, acyl-CoA dehydrogenase/ oxidoreductase	1.46±0.15	1.06±0.00	1.31±0.10
IPI00544747 (AT1G03090.2)	MCCA, Isoform 1 of Methylcrotonoyl-CoA carboxylase subunit alpha, mitochondrial	1.35±0.19	1.22±0.05	1.12±0.08
IPI00531611 (AT4G34030.1)	MCCB, Methylcrotonoyl-CoA carboxylase beta chain, mitochondrial	1.32±0.11	1.05±0.02	1.26±0.10
IPI00516986 (AT2G26800.1)	Isoform 2 of Hydroxymethylglutaryl-CoA lyase, mitochondrial	1.46±0.10	1.20±0.00	1.19±0.08
IPI00521527 (AT1G54100.2)	ALDH7B4, Aldehyde dehydrogenase family 7 member B4	1.40±0.01	1.29±0.11	1.07±0.13

IPI00542210 (AT1G54340.1)	ICDH, Isocitrate dehydrogenase	1.79±0.18	1.56±0.15	1.35±0.02
IPI00520407 (AT5G14800.1)	P5CR, Pyrroline-5-carboxylate reductase	1.37±0.11	1.00±0.05	1.28±0.06
**sucrose and starch metabolism**				
IPI00523295 (AT5G20830.2)	SUS1, Sucrose synthase 1	1.77±0.02	2.35±0.11	0.77±0.11
IPI00540190 (AT3G43190.1)	SUS4, UDP-glycosyltransferase/ sucrose synthase/ transferase, transferring glycosyl groups	2.68±0.17	3.12±0.04	0.80±0.13
IPI00524950 (AT5G37180.1)	SUS5, Sucrose synthase 5	1.16±0.09	1.65±0.27	0.73±0.20
IPI00530921 (AT1G73370.1)	SUS6, Sucrose synthase 6	1.27±0.13	1.53±0.25	0.88±0.10
IPI00541257 (AT1G78090.1)	TPPB, trehalose-6-phosphate phosphatase	1.48±0.14	1.03±0.15	1.41±0.01
**phenylpropanoid biosynthesis**				
IPI00519512 (AT2G37040.1)	PAL1, Phenylalanine ammonia-lyase 1	1.49±0.19	0.87±0.15	1.64±0.08
IPI00525638 (AT3G53260.1)	PAL2, Phenylalanine ammonia-lyase 2	1.80±0.12	0.93±0.08	1.88±0.02
IPI00533524 (AT3G10340.1)	PAL4, Phenylalanine ammonia-lyase 4	1.34±0.03	0.96±0.01	1.34±0.03
IPI00531924 (AT5G06720.1)	PA2, Peroxidase 53	1.99±0.01	0.79±0.18	2.53±0.16
IPI00548831 (AT1G49570.1)	Peroxidase 10	2.65±0.29	1.05±0.28	2.50±0.04
IPI00544435 (AT2G18150.1)	Peroxidase 15	1.33±0.17	_	_
IPI00518620 (AT3G32980.1)	Peroxidase 32	2.11±0.27	_	2.10±0.19
IPI00522050 (AT3G49120.1)	PRXCB, Peroxidase 34	2.46±0.05	1.15±0.26	2.18±0.18
IPI00545315 (AT4G08770.1)	Peroxidase 37	2.21±0.14	_	1.94±0.21
IPI00522834 (AT4G08780.1)	Peroxidase 38	2.13±0.09	1.29±0.29	1.75±0.19
IPI00539387 (AT4G33420.1)	Peroxidase 47	1.37±0.08	0.97±0.25	1.56±0.19
IPI00535466	Peroxidase 50	1.75±0.28	_	1.42±0.16

(AT4G37520.1)				
IPI00528868 (AT5G06730.1)	Peroxidase 54	2.90±0.01	1.18±0.14	2.52±0.00
IPI00527390 (AT5G19890.1)	Peroxidase 59	2.50±0.06	_	2.84±0.25
IPI00537511 (AT5G64120.1)	Peroxidase 71	1.53±0.28	_	1.85±0.01
**tricarboxylic acid (TCA) cycle**				
IPI00526152 (AT4G37870.1)	PCK1, Phosphoenolpyruvate carboxykinase [ATP]	1.32±0.03	1.20±0.00	1.06±0.02
IPI00540440 (AT5G65690.1)	PCK2, ATP binding phosphoenolpyruvate carboxykinase 2	1.34±0.01	0.88±0.14	1.60±0.18
IPI00542210 (AT1G54340.1)	ICDH, Isocitrate dehydrogenase	1.79±0.18	1.56±0.15	1.35±0.02
IPI00541202 (AT5G65750.1)	2-oxoglutarate dehydrogenase, E1 subunit	1.54±0.12	1.35±0.13	1.09±0.00
IPI00530824 (AT5G08300.1)	Succinyl-CoA ligase [ADP-forming] subunit alpha-1, mitochondrial	1.48±0.10	1.21±0.05	1.13±0.20
IPI00531652 (AT5G56720.1)	malate dehydrogenase, cytosolic, putative	1.39±0.03	1.38±0.06	0.98±0.02
IPI00526422 (AT1G10670.4)	ACLA-1, ATP-citrate synthase alpha chain protein 1	0.74±0.08	0.86±0.08	0.77±0.06
IPI00545433 (AT3G58750.1)	CSY2, Citrate synthase 2, peroxisomal	1.25±0.09	1.50±0.30	0.85±0.28
IPI00526256 (AT3G55410.1)	2-oxoglutarate dehydrogenase E1 component, putative / oxoglutarate decarboxylase, putative / alpha-ketoglutaric dehydrogenase, putative	1.19±0.19	1.34±0.25	0.88±0.10
IPI00526634 (AT1G09430.1)	ACLA-3, ATP-citrate synthase alpha chain protein 3	1.29±0.12	1.36±0.15	0.92±0.04
IPI00541759 (AT4G35260.1)	IDH1, Isocitrate dehydrogenase [NAD] regulatory subunit 1, mitochondrial	0.62±0.18	0.68±0.13	0.88±0.09

IPI00536659 (AT2G17130.2)	IDH2, Isoform 2 of Isocitrate dehydrogenase [NAD] regulatory subunit 2, mitochondrial	0.61±0.24	0.61±0.17	0.97±0.05
IPI00543566 (AT1G53240.1)	mMDH1, Malate dehydrogenase 1, mitochondrial	0.61±0.22	0.52±0.28	1.14±0.12
IPI00517821 (AT3G27380.2)	SDH2-1, Succinate dehydrogenase [ubiquinone] iron-sulfur subunit 1, mitochondrial	0.79±0.17	0.70±0.20	1.10±0.01

mitochondrial protein

IPI00538946 (ATMG00990.1)	NAD3, NADH-ubiquinone oxidoreductase chain 3	1.35±0.01	-	0.84±0.29
IPI00521971 (ATMG00480.1)	ORFB ATP synthase protein YMF19	1.65±0.13	2.05±0.21	0.81±0.10
IPI00785805 (ATMG01190.1)	ATP1, ATP synthase subunit alpha, mitochondrial	0.80±0.00	1.08±0.20	0.76±0.22
IPI00518390 (ATMG00510.1)	NAD7, NADH dehydrogenase [ubiquinone] iron-sulfur protein 2	1.22±0.12	1.42±0.13	0.86±0.09
IPI00544183 (ATMG00220.1)	COB Cytochrome b	1.53±0.04	1.57±0.05	1.02±0.08
IPI00520130 (AT2G07698.1)	ATP synthase alpha chain, mitochondrial, putative	1.02±0.03	1.33±0.03	0.83±0.13
IPI00535084 (ATMG01320.1)	NAD2B;NAD2A NADH-ubiquinone oxidoreductase chain 2	1.44±0.20	1.32±0.12	1.06±0.07
IPI00529948 (ATMG00070.1)	NAD9, NADH dehydrogenase [ubiquinone] iron-sulfur protein 3	1.28±0.22	1.24±0.23	0.98±0.03
IPI00527727 (ATCG01130.1)	ycf1;YCF1.2;YCF1.1 Putative membrane protein ycf1	1.37±0.18	1.29±0.19	1.02±0.02
IPI00529181 (ATMG00160.1)	COX2, Cytochrome c oxidase subunit 2	1.29±0.07	1.34±0.11	0.96±0.09
IPI00542419 (ATMG00665.1)	NAD5B;NAD5A;NAD5C; nad5 NADH-ubiquinone oxidoreductase chain 5	1.46±0.17	1.38±0.15	0.97±0.09

IPI00544671 (ATMG00090.1)	RPS3, Ribosomal protein S3, mitochondrial	_	_	1.29±0.07
IPI00542639 (ATMG00640.1)	ORF25, ATP synthase protein MI25	2.52±0.05	2.19±0.12	1.16±0.19
IPI00535114 (ATCG00490.1)	RBCL, Ribulose bisphosphate carboxylase large chain	_	0.80±0.14	_
